# Blocking TRPV4 Ameliorates Osteoarthritis by Inhibiting M1 Macrophage Polarization via the ROS/NLRP3 Signaling Pathway

**DOI:** 10.3390/antiox11122315

**Published:** 2022-11-23

**Authors:** Heng Sun, Ziying Sun, Xingquan Xu, Zhongyang Lv, Jiawei Li, Rui Wu, Yuxiang Fei, Guihua Tan, Zizheng Liu, Yuan Liu, Dongquan Shi

**Affiliations:** State Key Laboratory of Pharmaceutical Biotechnology, Department of Sports Medicine and Adult Reconstructive Surgery, Affiliated Drum Tower Hospital, Medical School, Nanjing University, 321 Zhongshan Road, Nanjing 210008, China

**Keywords:** osteoarthritis (OA), TRPV4, M1 macrophage polarization, NLRP3, reactive oxygen species (ROS)

## Abstract

Osteoarthritis (OA) is a low-level inflammatory disease in which synovial macrophage M1 polarization exacerbates the progression of synovitis and OA. Notedly, the ROS (reactive oxygen species) level in macrophages is intimately implicated in macrophage M1 polarization. TRPV4 (transient receptor potential channel subfamily V member 4), as an ion channel, plays a pivotal role in oxidative stress and inflammation. In this study, we investigated the role of TRPV4 in OA progression and M1 macrophage polarization. Male adult Sprague–Dawley (SD) rats underwent a medial meniscus radial transection operation to create an OA model in vivo and RAW 264.7 cells were intervened with 100 ng/mL LPS (lipopolysaccharide) to induce M1-polarized macrophages in vitro. We demonstrated that the infiltration of M1 synovial macrophages and the expression of TRPV4 were increased significantly in OA synovium. In addition, intra-articular injection of HC067074 (a specific inhibitor of TRPV4) alleviated the progression of rat OA and significantly decreased synovial macrophage M1 polarization. Further mechanisms suggested that ROS production by M1 macrophages was decreased after TRPV4 inhibition. In addition, NLRP3 (pyrin domain containing protein 3) as a downstream effector of ROS in M1-polarized macrophage, was significantly suppressed following TRPV4 inhibition. In conclusion, this study discovered that inhibition of TRPV4 delays OA progression by inhibiting M1 synovial macrophage polarization through the ROS/NLRP3 pathway.

## 1. Introduction

Osteoarthritis (OA) is the primary reason for disability and pain in people over 60 years of age [1]. It has been regarded as a complicated disease involving the degeneration of cartilage, formation of osteophytes, and inflammation of the synovium (synovitis) [2]. Synovitis is associated with aggravated chondrocyte apoptosis and hypertrophy, ectopic bone formation, and clinical symptoms, such as joint swelling and inflammatory pain [2]. Thus, focusing on the role of synovitis as an innate immune response might be a key to inhibiting the progression of OA.

The intimal lining layer of the normal synovium has two or three layers of synoviocytes, including fibroblasts and macrophages [3]. Synovial macrophages are activated during the development of OA, and these activated macrophages are categorized as either conventionally activated macrophages (M1 macrophages) or alternatively activated macrophages (M2 macrophages) due to different microenvironmental stimulations [4]. Recent research has found that M1 macrophages are recruited to the OA synovium and participate in OA symptoms of pain, joint space narrowing, and osteophyte formation by secreting numerous inflammatory cytokines and mediators, such as CXC chemokine ligand-10 (CXCL10), interleukin-1 (IL-1), interleukin-6 (IL-6), tumor necrosis factor-α (TNF-α), inducible nitric oxide synthase (iNOS), and cyclooxygenase-2 (COX-2) [5,6,7]. Consequently, a strategy for inhibiting M1 macrophage polarization still needs to be explored.

TRPV4 (transient receptor potential vanilloid 4), as a nonselective polymodal cation channel, is triggered by numerous stimuli (such as mechanical, temperature, and hypo-osmolarity) and can be blocked by pharmacological inhibitors [8,9]. The effect of TRPV4 on mechanical stimulation has been widely investigated in different diseases [10,11,12]. Mechanical stimulation or TRPV4 activation by the agonist GSK101 significantly increased matrix biosynthesis and anabolic gene expression of chondrocytes [13]. Furthermore, TRPV4 has been studied in pathological conditions for its participation in numerous macrophage functions, such as foam cell production and phenotypic switching [14,15]. In addition, recent research has shown that tissue stiffening could activate TRPV4 and increase the infiltration of M1 macrophages [15]. Interestingly, a previous study indicated that hypotonic stimulus resulted in ROS (reactive oxygen species) production through activation of the TRPV4 channel in synoviocytes [16]. Furthermore, ROS, as a crucial intracellular mediator for the activation of pro-inflammatory signaling pathways [17], favors the induction of the M1 macrophage polarization during OA synovitis onset and progression [18]. However, the role of TRPV4 in M1 synovial macrophage polarization remains ambiguous. Of note, the joint fluid of OA patients is hypotonic when compared to normal joint fluid [19]. However, whether TRPV4 participates in M1 synovial macrophage polarization by affecting ROS generation and the role of TRPV4 in synovitis remains to be explored.

In this study, M1 macrophage infiltration and TRPV4 expression were shown to be substantially elevated in the human OA synovium. In a rat OA model induced by medial meniscus radial transection (MMT), intra-articular administration of HC067047, a selective TRPV4 inhibitor, reduced synovial macrophage M1 polarization and decreased synovial inflammation, cartilage degradation, and osteophyte formation. Additionally, blocking TRPV4 decreased M1 macrophage polarization via the ROS (reactive oxygen species)/NLRP3 (pyrin domain containing protein 3) signaling pathway. According to our results, TRPV4 appears to be a unique therapeutic target for alleviating OA by relieving synovitis.

## 2. Materials and Methods

### 2.1. Human Tissues

Normal synovium (*n* = 3, 56–71 years old, 1 male, 2 females) was obtained from non-arthritic traffic-accident patients who underwent emergency amputation surgery within 1 day. OA synovium (*n* = 6, 62–73 years old, 2 males, 4 females, Kellgren–Lawrence grade = 4) was collected from total knee arthroplasty surgery cases because of serious OA. Synovium was preserved with 4% paraformaldehyde (PFA) for histological investigation. All human tissues were authorized and reviewed by the Ethical Committee of the Nanjing Drum Tower Hospital, the Affiliated Hospital of Nanjing University Medical School (2020-156-01).

### 2.2. Animal Experiments

All experiments were approved and carried out by the Animal Care and Use Committee of Nanjing Drum Tower Hospital, Nanjing University’s Affiliated Hospital (2021AE02001). All applicable ethical requirements were followed. The Nanjing Medical University Animal Center (Nanjing, China) provided male adult Sprague–Dawley (SD) rats (2 months, n = 24) and were randomly assigned to the sham, MMT, MMT + GSK101, or MMT + HC067047 groups. Following one week of adaptive feeding, 18 rats underwent a medial meniscus radial transection operation to create an OA model [20]. Six rats underwent a sham operation. Intra-articular injection of 50 µL of 5 μmol/L GSK101 (#HY-19608, MCE, New York, NY, USA) was administered twice per week in the MMT + GSK101 group, and 50 µL of 10 μmol/L HC067047 (#HY-100208, MCE, New York, NY, USA) was administered in the MMT + HC067047 group. The remaining groups received a 50 µL injection of phosphate-buffered saline (PBS). All of the rats were sacrificed after 8 weeks, and their hind legs were removed and preserved with 4% paraformaldehyde (PFA) for histological investigation and micro-CT scanning.

### 2.3. Cell Culture

RAW264.7 cells were procured through the Chinese Academy of Science’s Cell Bank of Type Culture Collection (Shanghai, China). The DMEM (Dulbecco’s modified Eagle’s medium, #C11995500BT Gibco, New York, NY, USA) was utilized to culture cells at 37 °C having 1% penicillin and streptomycin (#15140122, Gibco, New York, NY, USA) and 10% FBS (foetal bovine serum, #16000044 Gibco, New York, NY, USA). To induce M1-polarized macrophages, RAW 264.7 cells were intervened with 100 ng/mL LPS (lipopolysaccharide, #L2630, Sigma-Aldrich, St. Louis, MO, USA). To investigate the involvement of TRPV4 in M1 macrophage polarization, TRPV4 was activated with 5 μmol/L GSK101 and inactivated with 10 μmol/L HC067047. To activate NLRP3 inflammasome, the cells were pre-treated with LPS or LPS + HC067047 for 24 h, with 5 nmol/L ATP (#HY-B2176, MCE, New York, NY, USA) for 1 h. To study whether ROS participates in NLRP3 activation and macrophage polarization, the cells were incubated with LPS together with HC067047 or HC067047 + 20 μmol/L TBHP (#MKCH9944, Sigma, St. Louis, MO, USA) for 24 h.

### 2.4. Histological Analysis

The complete knees were fixed with 4% PFA (paraformaldehyde) for 48 h, then the joints were decalcified for 2 months in a 10% EDTA (Ethylene Diamine Tetraacetie Acid, #1430, Biofroxx, Guangzhou, China) solution and paraffin-embedded. After staining the coronal sections (3 μm) with HE (hematoxylin and eosin, #C0105S, Beyotime, Shanghai, China) and SO (safranin O/Fast Green, #G1371, Solarbio, Beijing, China), the slides were examined by independent double-blinded investigators to determine the synovitis and Osteoarthritis Research Society International (OARSI) scores. To determine M1 macrophage polarization, the synovium was stained with immunofluorescence.

### 2.5. Microcomputed Tomography (Micro-CT)

A micro-CT scanner (mCT80, Scanco Medical AG, Wangen-Brüttisellen, Switzerland) with a current of 114 μA, a voltage of 70 kV, and a resolution of 15.6 μm per pixel, was used to analyse the microstructure of the complete knee joints, as described previously [21]. Scanco Medical software was used to acquire the reconstruction images.

### 2.6. Quantitative Real-Time PCR

The total RNA extraction kit (#RN001, ES Science, Shanghai, China) was used for extracting cellular mRNA from RAW264.7 cells (6-well plates, approximately 6 × 10^5^ cells/well). Reverse transcription reagents (#R223-01, Vazyme Biotech, Piscataway, NJ, USA) were used for acquiring complementary DNA (cDNA) from mRNA. The cDNA in a 20 μL SYBR green qPCR kit (#Q411-02, Vazyme Biotech, Piscataway, NJ, USA) according to the protocol, were performed on a LightCycler 480 II (Roche, Basel, Switzerland). The primer sequences for genes tested with RAW264.7 cells were shown in Table 1, and GAPDH was the reference gene. The comparative threshold cycle (Ct) technique (2^−∆∆Ct^ method) was used to calculate the relative fold gene expression of samples [22].

### 2.7. Immunohistochemistry and Immunofluorescence

Endogenous oxidase was inactivated with 3% H_2_O_2_ (hydrogen peroxide) for 20 min after the sections were dewaxed in dimethyl benzene 2 times and soaked in graded ethanol. Then, the sections were antigen-retrieved with 0.2% pepsin (#P-700, Sigma-Aldrich, St. Louis, MO, USA), blocked for 45 min at 37 °C with goat serum (#16210072, Gibco, New York, NY, USA), then probed with 1:200 primary antibodies (4 °C, overnight). After that, an HRP-anti-IgG (#BL001A, Biosharp, Hefei, China) with a dilution of 1:5000, the secondary antibody, was used to incubate the sections, and 3,3-diaminobenzidine (#1205250, Typing, Shanghai, China) was used for visualizing the positive cells. The sections were examined and imaged with an optical microscope (Zeiss, Jena, Germany). Three areas of each section were randomly selected to count the number of positive cells and total cells, then calculate the positive cell rate. In the immunofluorescence experiment, following secondary antibody treatment and DAPI (4,6-diamidino-2-phenylindole, #AB104139, Abcam, Cambridge, Britain) staining of the nuclei, the sections were imaged under fluorescence microscopy (Zeiss, Jena, Germany). The method of calculating the rate of positive cell was the same as immunohistochemical. In cytological immunofluorescence experiment, after being fixed with 4% PFA (15 min), RAW264.7 cells in 24-well plates (approximately 1.0 × 10^5^ cells/well) were exposed to 0.1% Triton X-100 (15 min) for permeabilization, then blocked with 5% BSA (bovine serum albumin) for 1 h. After that, the cells were treated with 1:200 primary antibodies. The procedures described below are the same as those for immunofluorescence.

### 2.8. Western Blotting

RAW264.7 cells in 6-well plates (approximately 6 × 10^5^ cells/well) were treated for 15 min at 4 °C in RIPA (#R0010, Solarbio, Beijing, China) lysis buffer with 1 mmol/L protein phosphatase inhibitor (#P1260, Solarbio, Beijing, China) and 1 mmol/L phenylmethanesulfonyl fluoride (#329-98-6, Solarbio, Beijing, China). The lysates were extracted post-centrifuging step (4 °C, 12,000 r/min, 15 min). SDS-PAGE (SDS-polyacrylamide gels, 10%, EpiZyme, Shanghai, China) was used to separate proteins (20 µg per lane) before they were transferred to PVDF (polyvinylidene fluoride, Bio-Rad, Hercules, CA, USA) membranes. The blots were immersed in 5% BSA for 1 h before being incubated with 1:1000 primary antibodies at 4 °C overnight. After that, secondary antibodies with a dilution of 1:5000 were used to probe the blots for 1 h at 37 °C. The ChemiDocXRS + Imaging System (Tanon, Shanghai, China) was used to detect all of the images. Finally, ImageJ (NIH, Bethesda, MD, USA) was used to analyze the protein greyscale values.

### 2.9. Antibodies

The antibodies used were: rabbit anti-TRPV4 (#ab39260, Abcam, Cambridge, Britain); rabbit anti-iNOS (#13120, Cell Signaling Technology, Danvers, MA, USA); mouse anti-F4/80 (#sc-377009, Santa Cruz, Santa Cruz, CA, USA); rabbit anti-COX2 (#ab15191, Abcam, Cambridge, Britain); rabbit anti-GAPDH (#8884, Cell Signaling Technology, Danvers, MA, USA); rabbit anti-NLRP3 (#13158, Cell Signaling Technology, Danvers, MA, USA); rabbit anti-CASPASE-1 (#22915-1-AP, Proteintech, Wuhan, China); and rabbit anti-ASC (#10500-1-AP, Proteintech, Wuhan, China).

### 2.10. ELISA (Enzyme-Linked Immunosorbent Assay)

IL-6 ELISA Kit (#KE10007, Proteintech, Wuhan, China) and TNF-α ELISA Kit (#KE10002, Proteintect, Wuhan, China), directed by the manufacturer, were used to quantify IL-6 and TNF-α in the supernatant of RAW264.7 cells (6-well plates, approximately 6 × 10^5^ cells/well) respectively. Absorbances at 450 nm were read in a microplate reader (ThermoFisher, Waltham, MA, USA). IL-6 and TNF-α concentrations were normalized to the total cellular counts.

### 2.11. Measurement of Intracellular ROS Levels

RAW264.7 cells plated in 6-well plates (approximately 6.0 × 10^5^ cells/well) were stimulated by LPS for 6 h with HCO67047 or HC067047 + 20 μmol/L TBHP. The intracellular ROS levels were then visualized by incubating the cells with DCFH-DA (2′,7′-dichlorodihydrofluorescein diacetate, #S0033S, Beyotime, Shanghai, China) at 37 °C for 30 min. Fluorescence microscopy (Zeiss, Jena, Germany) performed imaging of the cells. ImageJ (NIH, Bethesda, MD, USA) was used to measure the fluorescence intensity of images.

### 2.12. Measurement of Mitochondrial Membrane Potential (JC-1 Staining)

RAW264.7 cells in 6-well plates (approximately 3.0 × 10^5^ cells/well) were stimulated by LPS for 6 h with or without HC067047. A mitochondrial membrane potential assay kit with JC-1 (#C2003S, Beyotime, Shanghai, China) was used to detect the change of mitochondrial membrane potential. The cells in each treatment group were washed in PBS before being given 1 mL DMEM and 1 mL JC-1 (1X). After that, the cells were cultured for 20 min at 37 °C then rinsed with JC-1 dyeing buffer. A fluorescence microscope (Zeiss, Jena, Germany) was used to obtain all of the images. JC-1 red indicates normal mitochondrial membrane potential, while JC-1 green fluorescence shows decreased mitochondrial membrane potential.

### 2.13. Lactate Measurement

RAW264.7 cells in 6-well plates (approximately 6.0 × 10^5^ cells/well) were stimulated by LPS for 6 h with or without HC067047. A lactic acid assay kit (SNM184, Biolab, Beijing, China) was used to quantify lactate concentration in the supernatant of the cells, directed by the manufacturer. Absorbances at 530 nm were read in a microplate reader (ThermoFisher, Waltham, MA, USA). Standard preparation (3 mmol/L) was used as reference.

### 2.14. Statistical Analysis

The quantitative data were collected through independent experiments repeated 3 times and statistically analyzed with GraphPad Prism 8.0. Comparing the means of three or more groups required a one-way analysis of variance (ANOVA) with Tukey’s post-hoc test, whereas a test of independence (*t*-tests, unpaired two-tailed) was employed to examine differences between the two groups. If *p* < 0.05 (* *p* < 0.05, ** *p* < 0.01, *** *p* < 0.001, **** *p* < 0.001), it was regarded to be statistically significant. Data are shown as mean ± SD.

## 3. Results

### 3.1. M1 Macrophages and TRPV4 Expression Are Highly Elevated in OA Synovium

**Figure 1:**
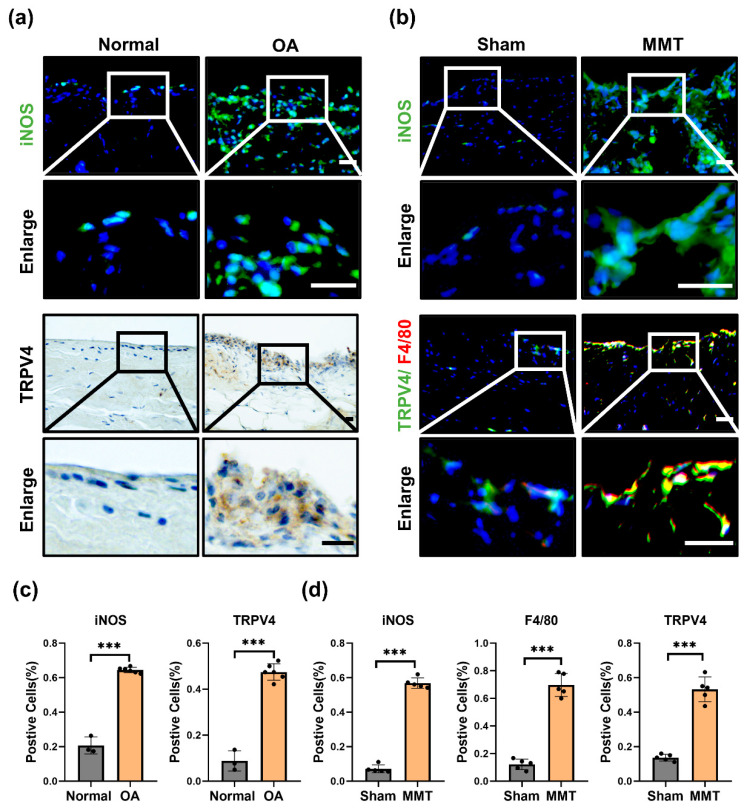
TRPV4 and M1 macrophages are elevated in OA synovium. (**a**) Immunofluorescence images of iNOS and immunohistochemical staining of TRPV4 in normal and OA human synovium (Scale bar: 50 μm). (**b**) Immunofluorescence images of iNOS, TRPV4, and F4/80 in the sham and MMT rat synovium (Scale bar: 50 μm). (**c**) Percentages of iNOS- and TRPV4-positive cells in human synovium. normal human synovium sample = 3, OA human synovium sample = 6. Unpaired two-tailed *t*-test, *** *p* < 0.001. (**d**) Percentages of iNOS-, F4/80-, and TRPV4-positive cells in sham and MMT group. n = 5. Unpaired two-tailed *t*-test, *** *p* < 0.001. All data are shown as mean ± SD.

To investigate whether TRPV4 participates in M1 macrophage polarization and OA progression. We investigated the expression of TRPV4 and an M1 macrophage marker (inducible nitric oxide synthase, iNOS) in the human and rat OA model (MMT-induced OA rat model) synovium. Compared to the normal synovium, the quantity of iNOS-positive cells was greatly elevated in OA humans (Figure 1a,c). Furthermore, with the upregulated infiltration of M1 macrophages, the rate of TRPV4-positive cells was considerably increased in the synovium of OA humans (Figure 1a,c). Similar to the human OA synovium, the infiltration of M1 macrophages (iNOS^+^) was aggravated in the synovium of the MMT groups, accompanied by a considerably higher proportion of TRPV4-positive cells (Figure 1b,d). F4/80, as a marker of macrophages, has been widely used in studies about macrophages [23]. Of note, TRPV4 was located mostly in the accumulated macrophages, with colocalization of TRPV4 and F4/80 (Figure 1b). These findings implied that M1 macrophages accumulate accompanied by dramatically elevated TRPV4 expression in OA synovium.

### 3.2. Inhibition of TRPV4 Decreased the Expression Levels of M1 Phenotypic Markers

**Figure 2:**
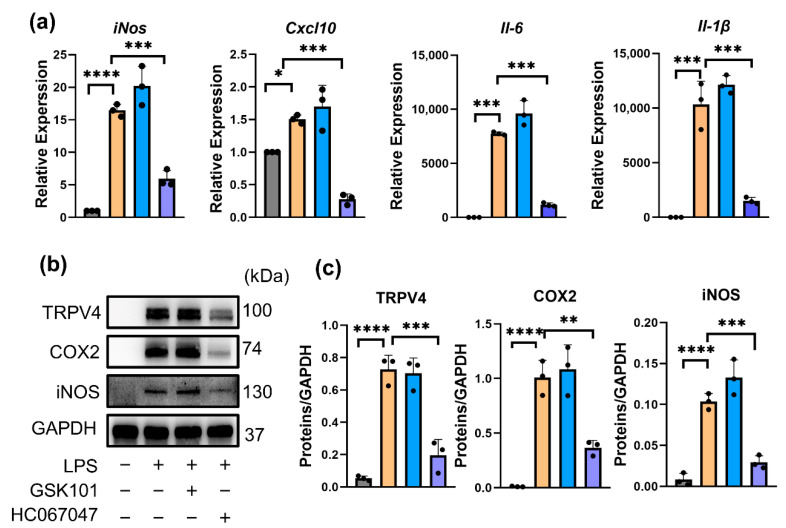

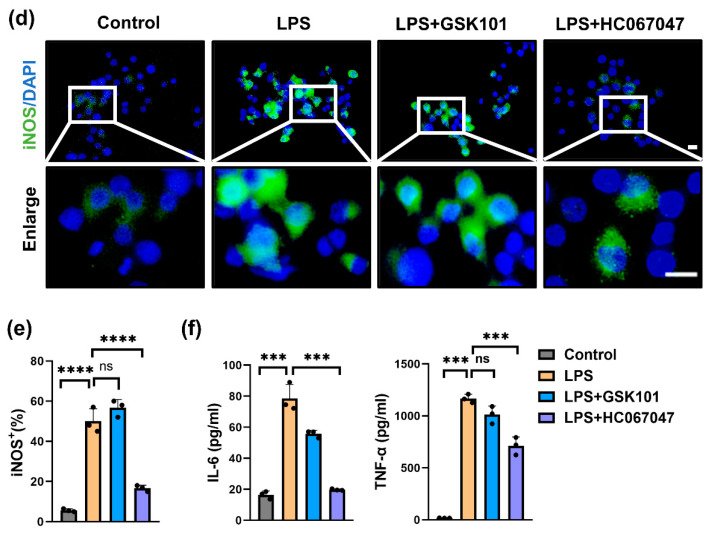
Inhibition of TRPV4 decreased the expression levels of M1 phenotypic markers. (**a**) *iNos, Il-6, Cxcl-10,* and *Il-1β* mRNA levels in RAW26.47 cells after 100 ng/mL LPS treatment with 5 μmol/L GSK101 or 10 μmol/L HC067047 for 6 h. n = 3. One-way ANOVA with Tukey’s post-hoc test, * *p* < 0.05; *** *p* < 0.001, ****p < 0.0001. (**b**) Protein levels of TRPV4, COX2, and iNOS in the cells after 100ng/mL LPS treatment with 5 μmol/L GSK101 or 10 μmol/L HC067047 for 24 h. (**c**) Quantitation of Western blotting; n = 3. One-way (ANOVA) with Tukey’s post-hoc test, ** *p* < 0.01; *** *p* < 0.001, **** *p*< 0.0001. (**d**) Immunofluorescence analysis of iNOS in the cells after 24 h (Scale bar: 10 μm). (**e**) Quantitation of (**d**). n = 3. One-way ANOVA with Tukey’s post-hoc test, **** *p*< 0.0001; ns = no significance. (**f**) The ELISA assay was used to determine the levels of IL-6 and TNF-α in the supernatants collected after 24 h. n = 3. One-way ANOVA with Tukey’s post-hoc test, *** *p* < 0.001, ns = no significance. All data are shown as mean ± SD.

HC067047, as a potent (IC_50_ = 17 nmol/L) and selective TRPV4 antagonist, could reversibly inhibit currents through TRPV4 [24], and GSK101 is a selective agonist of TRPV4 [25]. To uncover whether targeting TRPV4 can inhibit M1 macrophage polarization in vitro, RAW264.7 cells were stimulated by 100 ng/mL LPS, a model of M1 macrophages, with or without 10 μmol/L HC067047 or 5 μmol/L GSK101. Compared to vehicle-treated cells, *iNos, Il-6, Cxcl10,* and *Il-1β* mRNA levels (Figure 2a) and iNOS, COX2, and TRPV4 protein levels (Figure 2b,c) were significantly elevated in LPS-treated cells (M1 macrophages). This finding indicated that TRPV4 participates in LPS-induced M1 macrophage polarization. Furthermore, qPCR and Western blot analysis demonstrated that suppression of TRPV4 by HC067047 result in a considerable decrease in the mRNA and protein levels of M1 phenotypic markers (Figure 2a–c), but activation of TRPV4 by GSK101 did not change the phenotype of M1 macrophages (Figure 2a–c). Immunofluorescence results also showed reduced expression of iNOS in HC067047-treated M1 macrophages (Figure 2d,e). At the same time, ELISA analysis detected that the protein levels of IL-6 and TNF-α in M1 macrophage supernatants were dramatically decreased when TRPV4 was inhibited. (Figure 2f). All of these results highlighted that TRPV4 inhibition suppressed the generation of proinflammatory factors in LPS-treated macrophages (M1 macrophages).

### 3.3. Blocking TRPV4 Alleviates OA by Suppressing M1 Macrophage Polarization In Vivo

**Figure 3:**
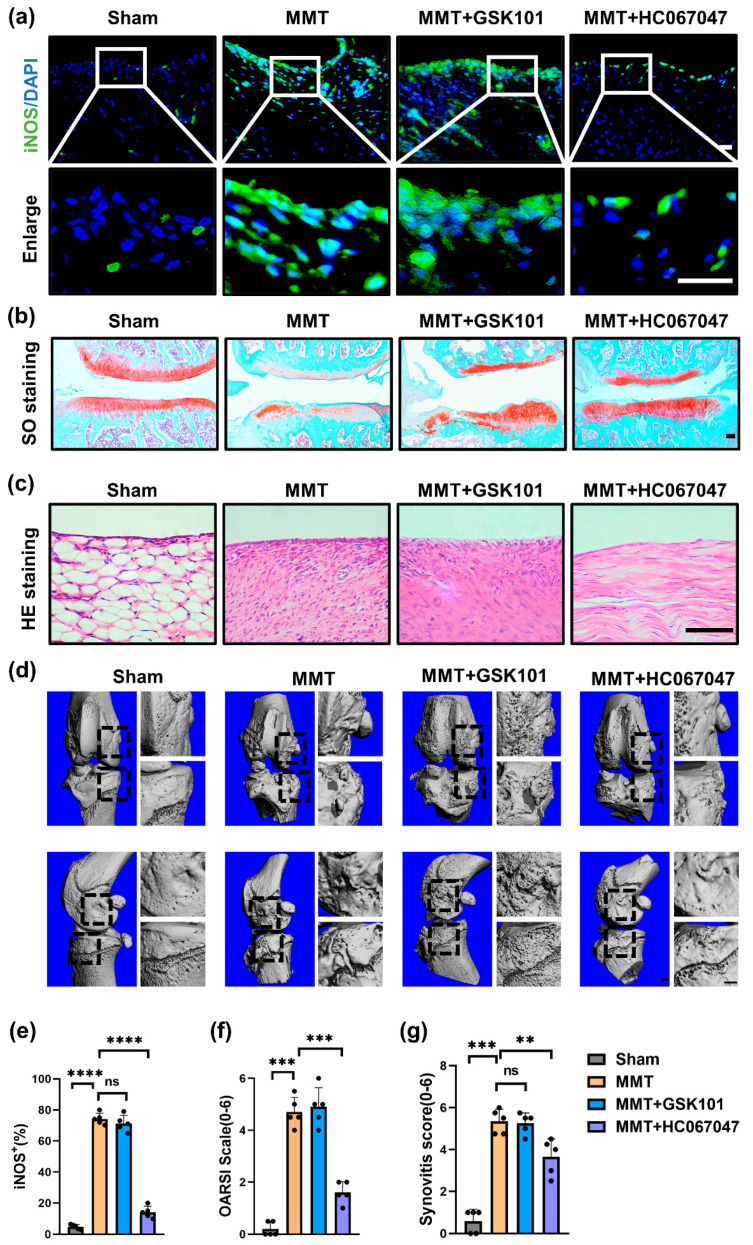
TRPV4 blockade alleviates OA by targeting M1 macrophages. (**a**) Immunofluorescence of iNOS in the synovium of the sham, MMT, MMT + GSK101, and MMT + HC067047 group. (Scale bars: 10 μm). (**b**) SO (safranin O/Fast Green) staining of knee joints from the sham, MMT, MMT + GSK101, and MMT + HC067047 groups (Scale bars: 100 μm). (**c**) HE staining of synovium from each of the 4 groups (Scale bars: 100 μm). (**d**) Images of reconstructed knee joints from each study group (Scale bars: 100 μm) (**e**) Percentages of iNOS-positive cells of animal sample. n = 5. One-way ANOVA with Tukey’s post-hoc test, **** *p* < 0.0001; ns = no significance. (**f**) Analysis of the OARSI score quantitatively in the 4 groups. n = 5. One-way ANOVA with Tukey’s post-hoc test, *** *p* < 0.001. (**g**) Synovitis grade quantification. n = 5. One-way ANOVA with Tukey’s post-hoc test, ** *p* < 0.01; *** *p* < 0.001; ns = no significance. All data are shown as mean ± SD.

To study the role of TRPV4 in M1 synovial macrophages in OA, MMT surgery was used to induce a rat OA model [26], and inhibition of TRPV4 alleviated OA and inhibited M1 synovial macrophage polarization by intra-articular administration of HC067047. After 8 w of MMT surgery, mice acquired the pathological characteristics of OA, involving breakdown of cartilage, inflammation of the synovium (synovitis), and formation of osteophytes (Figure 3b–d). However, compared to the MMT group, the MMT + HC067047 group showed less infiltration of M1 macrophages (iNOS^+^) in the synovium (Figure 3a,e), indicating that TRPV4 inhibition suppresses M1 synovial macrophage polarization in vitro. Furthermore, consistent with the decrease in M1 macrophages, cell infiltration of the synovium was also reduced following HC067047 treatment (Figure 3c). Analysis of representative images of the cartilage extracellular matrix revealed that inhibition of TRPV4 could delay the breakdown of cartilage in the MMT-induced OA rat model (Figure 3b). The synovitis score and OARIS scores both decreased significantly (Figure 3f,g). Moreover, osteophytes, a major feature of OA progression [27], decreased significantly after HC067047 administration (Figure 3d). In conclusion, TRPV4 suppression caused by intra-articular administration of HC067047 could prevent M1 synovial macrophage polarization and OA development in vivo.

### 3.4. TRPV4 Inhibition Prevented M1 Macrophage Polarization by Suppressing the NLRP3 Inflammasome

**Figure 4:**
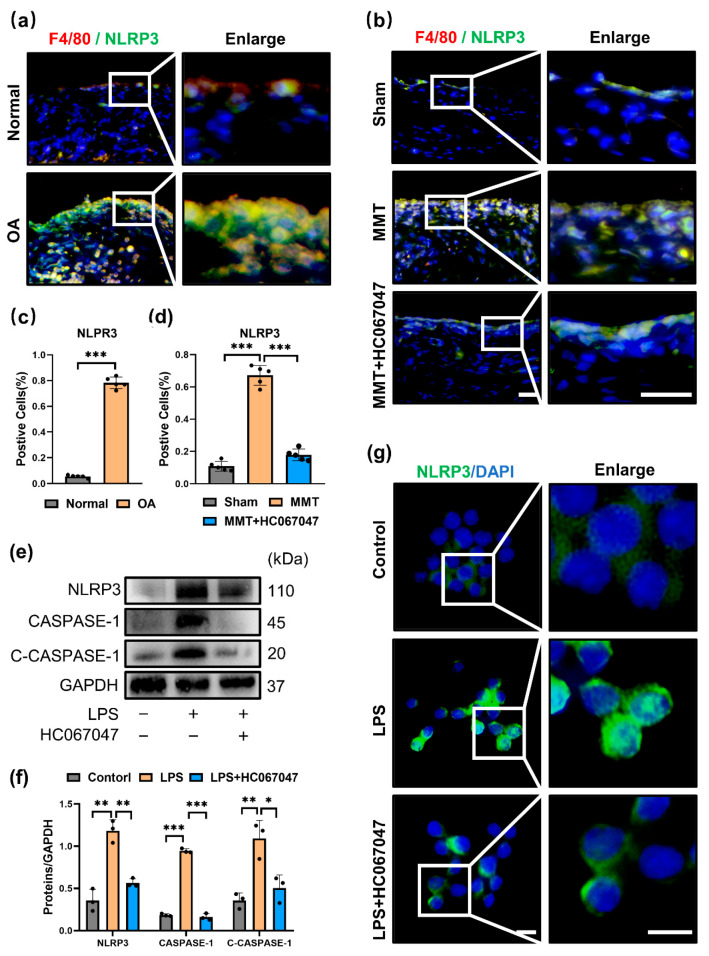
TRPV4 blockade inhibits the NLRP3 inflammasome (**a**) NLRP3 and F4/80 co-immunofluorescence in the normal and OA human synovium. (**b**) NLRP3 and F4/80 co-immunofluorescence in the synovium of the sham, MMT, and MMT + HC067047 groups (Scale bar: 50 μm). (**c**) NLRP3-positive cell quantification in human synovium. normal human synovium sample = 3, OA human synovium sample = 6. Unpaired two-tailed *t*-test, *** *p* < 0.001. (**d**) NLRP3-positive cell quantification of animal sample. n = 5. One-way ANOVA with Tukey’s post-hoc test, *** *p* < 0.001. (**e**) Western blot analysis of NLRP3, CASPASE-1, and cleaved CASPASE-1 (C-CASPASE-1) in the cells after intervention with LPS with or without HC067047 for 24 h. (**f**) Quantitation of Western blotting; n = 3. One-way ANOVA with Tukey’s post-hoc test, * *p* < 0.05; ** *p* < 0.01; *** *p* < 0.001. (**g**) Immunofluorescence staining of NLRP3 in the cells. All data are shown as mean ± SD.

To further investigate the underlying mechanism of TRPV4 on M1 macrophage polarization. We evaluated whether blockade of TRPV4 can inhibit the NLRP3 inflammasome, both priming and activating signals are required, which is the main characteristic of the M1 macrophage phenotype [28,29]. Compared to the normal group, NLRP3 was significantly elevated in the synovium of human OA and mostly colocalized with macrophage markers (F4/80) (Figure 4a,c). Consistent with human OA synovium, NLRP3 colocalized with macrophages (F4/80^+^) was markedly increased (Figure 4b,d) in the MMT group. However, blockade of TRPV4 by injection of HC067047 showed that the expression of NLRP3 in synovial macrophages was markedly reduced (Figure 4b,d).

In vitro, coinciding with previous studies, the priming and activation of NLRP3 were significantly increased in LPS-treated macrophages [30,31], with elevated expression of NLRP3, CASPASE-1, and cleaved-CASPASE-1 (Figure 4e,f). However, with TRPV4 inhibition by HC067047, the expression of NLRP3 was obviously decreased in M1 macrophages, as shown by immunofluorescence (Figure 4g). Furthermore, Western blot analysis showed that the expression levels of NLRP3, CASPASE-1 and cleaved-CASPASE-1 were significantly reduced in M1 macrophages treated with HC067047 (Figure 4e,f). These results imply that the blockade of TRPV4 attenuates LPS-induced NLRP3 priming and activation.

**Figure 5:**
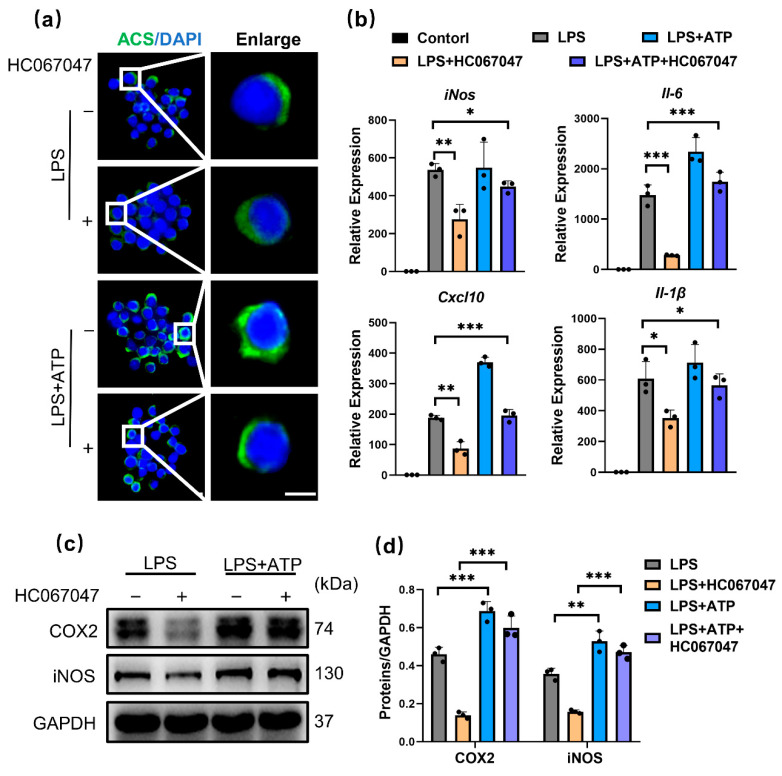
TRPV4 inhibition prevents M1 macrophage polarization by blocking the NLRP3 inflammasome. (**a**) Immunofluorescence of ASC in cells pretreated with 100ng/mL LPS or LPS + 10 μmol/L HC067047 for 6 h, without or with 5 nmol/L ATP for 1 h. (**b**) *iNos, Il-6, Cxcl-10,* and *Il-1β* mRNA levels in the control, LPS, LPS + HC067047, LPS + ATP, and LPS + ATP + HC067047 groups. n = 3. ANOVA with Tukey’s post-hoc test, * *p* < 0.05; ** *p* < 0.01; *** *p* < 0.001. (**c**) COX2 and iNOS protein levels in RAW264.7 cells pretreated with LPS or LPS + HC067047 for 24 h, without or with 2 nmol/L ATP for 3 h. (**d**) Quantitative analysis of Western blotting. n = 3. One-way ANOVA with Tukey’s post-hoc test, ** *p* < 0.01; *** *p* < 0.001. All data are shown as mean ± SD.

To investigate whether the NLRP3 inflammasome is required for TRPV4-mediated M1 macrophage polarization inhibition, ATP, which is an NLRP3 activator that promotes the flow of potassium ions [32], was used. Immunofluorescence analysis showed that ASC was polarized to one side of the perinuclear area, a feature of NLRP3 activation, in response to ATP stimulation in HC067046-treated M1 macrophages (Figure 5a). This finding indicated that ATP blocked the effect of HC067047 on the inhibition of the NLRP3 inflammasome. In addition, ATP also blocked the impact of HC067047 on suppressing the generation of proinflammatory factors (Figure 5b–d). According to these results, we suggest that TRPV4 suppression decreases M1 macrophage polarization by blocking the NLRP3 signaling pathway.

### 3.5. Blockage of TRPV4 Inhibited NLRP3 via Mitochondrial ROS

**Figure 6:**
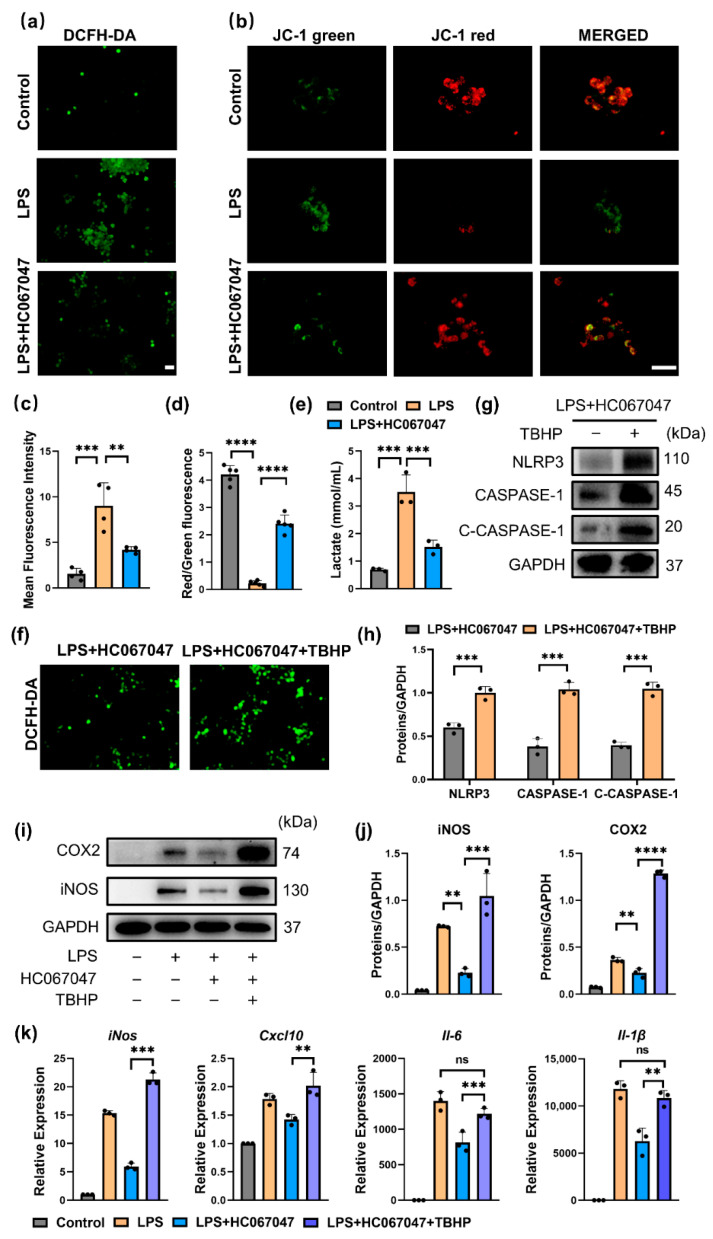
TRPV4 blockade inhibited NLRP3 via mitochondrial ROS. (**a**) DCFH-DA fluorescence of mitochondrial ROS in 100 ng/mL LPS-treated cells with 10 μmol/L HC067047 for 6 h (Scale bar: 50 μm) (**b**) JC-1 green and JC-1 red fluorescence and their colocalization in RAW264.7 cells (Scale bar: 50 μm). (**c**) Quantitative analysis of the MFI (mean fluorescence intensity) of DCFH-DA. n = 4. One-way ANOVA with Tukey’s post-hoc test, ** *p* < 0.01; *** *p* < 0.001. (**d**) Quantitative analysis of the ratio of JC-1 red to JC-1 green staining. n = 5. One-way ANOVA with Tukey’s post-hoc test, **** *p* < 0.001. (**e**) Lactate concentration in RAW 264.7 cells supernatant after stimulated with LPS with or without HC067047 for 6 h. n = 3. One-way ANOVA with Tukey’s post-hoc test *** *p* < 0.001. (**f**) DCFH-DA fluorescence in RAW264.7 cells stimulated with LPS + HC067047 with or without 20 μmol/L TBHP for 6 h. (**g**) Western blot analysis of NLRP3, CASPASE-1, and cleaved CASPASE-1 (C-CASPASE-1) in RAW264.7 cells after treatment with LPS + HC067047 with or without 20 μmol/L TBHP for 24 h. (**h**) Quantitative analysis of the protein level in (**g**). n = 3. One-way ANOVA with Tukey’s post-hoc test, *** *p* < 0.001. (**i**) COX2 and iNOS protein levels in the cells after treatment with LPS, LPS + HC067047, and LPS + HC067047 + 20 μmol/L TBHP for 24 h. (**j**) Quantitation of (**i**). n = 3. One-way ANOVA with Tukey’s post-hoc test, ** *p* < 0.01; *** *p* < 0.001; **** *p* < 0.0001. (**k**) *iNos, Il-6, Cxcl-10,* and *Il-1β* mRNA levels in cells after treatment with LPS, LPS + HC067047, LPS + HC067047 + 20 μmol/L TBHP for 6 h. n = 3. One-way ANOVA with Tukey’s post-hoc test, ** *p* < 0.01; *** *p* < 0.001; ns = no significance. All data are shown as mean ± SD.

Then, we investigated how TRPV4 affects mitochondrial dysfunction in M1 macrophages, which leads to NLRP3 inflammasome activation [33]. In our study, LPS stimulation (M1 macrophages) resulted in a considerable increase in mitochondrial reactive oxygen species (ROS) generation in RAW264.7 cells, as evidenced by immunofluorescence (Figure 6a,c). However, the HC067047 treatment considerably decreased ROS generation (Figure 6a,c). To determine whether the reduction in the accumulation of dysfunctional mitochondria was responsible for the reduction in mitochondrial ROS, JC-1 labeling was utilized to measure mitochondrial membrane potential [28]. Immunofluorescence analysis showed that HC067047 decreased the intensity of JC-1 green while increasing the fluorescence intensity of JC-1 red (Figure 6b,d), indicating that the mitochondrial membrane potential was stabilized following HC067047 treatment. In addition, we detected the level of lactate in cells treated with LPS with or without HC067047. Coinciding with previous studies, LPS stimulation of RAW 264.7 cells resulted in increased levels of lactate production [34]. Interestingly, this effect was reduced when TRPV4 was inhibited by HC067047 (Figure 6e). These results suggested that the production of ROS was considerably decreased in M1 macrophages by stabilizing the mitochondrial membrane potential and decreasing glycolysis when TRPV4 was inhibited.

To investigate the effect of ROS on the M1 macrophage polarization and NLRP3 inflammasome activation in vitro, TBHP (tert-butyl hydroperoxide), which promotes mitochondrial ROS production [35], was used in HC067047-treated M1 macrophages. The generation of mitochondrial ROS was significantly enhanced after TBHP treatment (Figure 6f), and the priming and activation of the NLRP3 inflammasome were also elevated following TBHP stimulation (Figure 6g,h). In addition, TBHP markedly increased the protein levels of COX2 and iNOS, and mRNA levels of *iNos, Il-6, Cxcl10,* and *Il-1β.* (Figure 6i–k). In summary, these results indicate that ROS mediates the role of HC067047 in inhibiting NLRP3 activation and M1 macrophage polarization.

## 4. Discussion

The TRPV channel family (TRPV1-6) is triggered by a variety of physiopathological environments including temperature, osmotic pressure, and mechanical stress [36,37,38]. It plays a pivotal role in inflammation [36,39,40,41]. In our previous studies, we identified TRPV1 channels as a therapeutic target to inhibit synovitis as well as OA progression [6]. In this study, we showed that the blockade of TRPV4 attenuated OA progression by inhibiting M1 macrophage polarization. Our results revealed that the cartilage damage, osteophytes formation, and synovitis, all of which were alleviated by HC067047 (a specific inhibitor of TRPV4) treatment, decreased the numbers of M1 macrophages in the synovium of rat OA. In addition, we discovered that the markers associated with M1 macrophages were significantly inhibited via the ROS/NLRP3 signaling pathway by HC067047 treatment in vitro.

OA, as a degenerative disease, was affected by a variety of factors such as genes, environment, mechanical stress, and inflammation [42]. TRPV4 was a vital cell-surface sensor that transduced mechanical and osmotic pressure to regulate the anabolism and catabolism of cells [43]. A previous study showed that TRPV4 was a possible sensor for excessive stress and resulted in chondrocyte apoptosis in OA [44]. In contrast, a recent study suggested that TRPV4 activation by GSK101 significantly increased the expression of collagen II and sulfated glycosaminoglycans (GAGs) in cartilage [13]. These data demonstrated the essential but unclear roles of TRPV4 in OA progression. Not only mechanical stress, but also synovitis, especially M1 macrophage polarization, received increasing attention [45]. According to clinical surveys and empirical findings, M1 macrophages are thought to be responsible for OA progression, including osteophyte generation and synovitis [46,47,48]. As a result, treatment techniques aimed at reducing the number of M1 macrophages or shifting M1 macrophages to M2 macrophages have been proposed as a way to attenuate OA progression. TRPV4, as an important member of the TRPV channel family, plays a pivotal role in oxidative stress and inflammation [40]. In a high tidal volume breathing-induced lung injury mouse model, pulmonary barrier permeability was reduced by suppressing TRPV4, which decreased inflammatory cytokine expression in M1 macrophages [49]. Furthermore, in an LPS-induced acute lung damage model, blocking TRPV4 function prevented pneumonia and decreased the generation of proinflammatory markers, including IL-6, TNF-α, and ROS [50]. Interestingly, compared to normal joint fluid, the joint fluid of OA patients is hypotonic which is the natural activation environment for TRPV4 [51]. However, whether TRPV4 participates in M1 synovial macrophage polarization in OA remains unknown. Here, we observed an increase in TRPV4 expression in the synovium of OA humans and MTT rats, accompanied by the upregulated infiltration of M1 macrophage. Furthermore, we discovered that the blockade of TRPV4 significantly decreased both the mRNA levels and the protein levels of proinflammatory factors in vitro. In addition to vitro research, we further showed that the blockade of TRPV4 reduced the density of M1 macrophages in OA rat synovium, as highlighted by the decreased numbers of iNOS-positive cells, which might explain how OA progression was attenuated. This study highlights the role of TRPV4 in macrophage and will contribute to a more comprehensive understanding of the therapeutic effect of targeting TRPV4 on OA.

The NOD-like receptor protein 3 inflammasome (NLRP3) aggravated the pathological conditions of numerous arthritis, including OA and gouty arthritis, by generating proinflammatory cytokine [52,53]. Previous studies have shown that the expression of NLRP3 is greatly upregulated in rat OA synovium. With MCC950 or CY-09 (two specific inhibitors of the NLRP3 inflammasome) injection, degradation and inflammation were decreased in the rat OA model [54,55]. Furthermore, in a model of TMAO (choline-metabolized trimethylamine N-oxide)-induced M1 polarization, both inhibiting NLRP3 and deleting the NLRP3 gene in macrophages resulted in a lower profile of M1 signature expression (*Il-1*, *Il-6*, *Cxcl10*, *Tnf-α*), demonstrating that NLRP3 activation is important for M1 macrophage polarization [28]. Of note, in the LPS-induced depression mouse model, the TRPV4 inhibitor HC067047 or TRPV4 shRNA could effectively rescue the abnormal behaviors by decreasing the expression of the NLRP3 [9]. However, the involvement of NLRP3 in M1 synovial macrophage polarization in OA remains unclear. Here, we found that NLRP3 was increased in the macrophages of human and rat OA synovium. Furthermore, NLRP3 expression was increased and partially increased in the perinuclear region in M1 macrophages in vitro. However, after treatment with HC067047 (an inhibitor of TRPV4), the expression of NLRP3 in the synovium of MMT model was markedly decreased. In addition, compared with that in the LPS group, NLRP3 activation was significantly inhibited in the HC067047-treated group in vitro, as shown by the lower protein levels of cleaved CASPASE-1 and the decrease in ASC polarization. These results supported the hypothesis that TRPV4 blockade affects the priming and activation of the NLRP3 inflammasome to inhibit M1 macrophage polarization.

Mitochondria-derived ROS were shown to be elevated in LPS-treated macrophages, which was associated with both NLRP3 activation and M1 macrophage polarization [9]. The latest research demonstrated that aerobic glycolysis was induced upon activation and the activities of the respiratory chain (oxidative phosphorylation) were attenuated in M1 macrophage, allowing for reactive oxygen species (ROS) production [56,57]. Similarly, a recent study found that the levels of lactate and ROS production were increased in LPS-induced M1 macrophage, indicating enhanced glycolysis [34]. TRPV4, as a mechanical sensor on the surface of cells, also plays a vital role in energy metabolism. Several previous studies have shown that ablation of TRPV4 inhibits ROS production in endothelial cells and immune cells by repairing the process of oxidative phosphorylation [58,59]. Our study supported their conclusion, LPS stimulation (M1 macrophages) resulted in a large elevation in mitochondrial reactive oxygen species (ROS) generation, whereas HC067047 treatment decreased the production of ROS. Furthermore, TBHP, which increases endogenous ROS generation through disrupting the function of mitochondria, increased NLRP3, CASPASE-1, and cleaved CASPASE-1 protein levels, in addition, blocked the effect of HC067047 on suppressing M1 macrophage polarization.

This study has several limitations. Firstly, we did not evaluate the effect of M1 macrophage inhibition by targeting TRPV4 on anabolism and catabolism of chondrocyte and synovioblast, which would impede a comprehensive understanding of the role of TRPV4 in OA. Secondly, although we found the ROS production was decreased in M1 macrophage by inhibiting TRPV4, the underlying mechanisms of them needs to be investigated.

## 5. Conclusions

In conclusion, we discovered that TRPV4 is a potential therapeutic target for preventing OA progression by suppressing M1 macrophage polarization and reducing synovial inflammation. TRPV4 inhibition mediated by HC067047 mechanically decreased M1 macrophage marker expression via the ROS/NLRP3 signaling pathway. Future research should focus on the underlying mechanism of how TRPV4 affects the ROS/NLRP3 pathway as a membrane protein.

## Figures and Tables

**Table 1:** The primer sequences and accession number of genes.

Gene	Accession Number	Forward	Reverse
*Il-1β*	NM_008361.4	5′-TGCCACCTTTTGACAGTGATG-3′	5′-TGCCACCTTTTGACAGTGATG-3′
*Il-6*	NM_031168.2	5′-TAGTCCTTCCTACCCCAATTTCC-3	5′-TTGGTCCTTAGCCACTCCTTC-3′
*iNos*	NM_001313922.1	5′-GCGCTCTAGTGAAGCAAAGC-3′	5′-AGTGAAATCCGATGTGGCCT-3′
*Cxcl10*	NM_021274.2	5′-CCACGTGTTGAGATCATTGCC-3′	5′-GAGGCTCTCTGCTGTCCATC-3′
*Gapdh*	NM_001289726.2	5′-AGGTCGGTGTGAACGGATTTG-3′	5′-TGTAGACCATGTAGTTGAGGTCA-3′

## Data Availability

Data are contained within the article.
